# 凝胶渗透色谱-气相色谱-离子阱质谱法测定桔梗原药和当归提取物中101种农药残留

**DOI:** 10.3724/SP.J.1123.2022.03042

**Published:** 2023-02-08

**Authors:** Rong ZHANG, Yue CHEN, Pei ZHENG, Ying DAI, Shasha LI, Yingyi JIA, Ran XIE, Jinhua WANG

**Affiliations:** 1.中国海关科学技术研究中心，北京 100026; 1. Science and Technology Research Centre of China Customs，Beijing 100026，China; 2.山东警察学院，山东 济南 250200; 2. Shandong Police College，Jinan 250200，China

**Keywords:** 凝胶渗透色谱, 气相色谱-离子阱质谱, 农药残留, 桔梗, 当归提取物, gel permeation chromatography （GPC）, gas chromatography-ion trap spectrometry （GC-ITMS）, pesticide residues, *Platycodonis radix*, extracts of *Angelica sinensis*

## Abstract

建立了凝胶渗透色谱（GPC）-气相色谱-离子阱质谱同时检测桔梗原药和当归提取物中101种农药残留的分析方法。方法采用乙腈超声辅助提取桔梗原药和当归提取物，浓缩提取液至近干后用乙酸乙酯-环己烷（1∶1， v/v）复溶，采用凝胶渗透色谱法（选取40 cm长、内径20 mm的凝胶渗透色谱柱）对样品进行净化，弃去前段含脂类、色素等杂质的流出液，收集17~30 min洗脱液并旋转蒸发浓缩至近干，甲苯1 mL定容上机。选用DB-5MS毛细管色谱柱分离待测物，通过离子阱质谱实现对101种农药残留的高效检测。方法通过优化前处理条件和离子阱二级质谱参数，有效降低了复杂中药基质对待测化合物的干扰，最大限度提高了样品中农药的定量准确性和回收率，101种农药3水平添加的平均回收率为58.3%~108.9%，每个添加水平10次独立重复测定的相对标准偏差为0.4%~16.5%，检出限（LOD）范围为0.2~40.0 μg/kg，可满足当前韩国、日本、欧洲规定的最大残留限量（maximum residue limits， MRLs）要求。方法具有操作简单快速、灵敏度高、重复性好等特点，凝胶渗透色谱技术的应用克服了固相萃取小柱净化容量不足的弊端，离子阱技术的应用可以进一步排除共流出基体杂质的干扰，提高定量和定性的准确性，检测效果优于常用的气相色谱-质谱法，是对中药中同时分析多种农药残留检测方法的有益补充。

## Abstract (en)

With the growing global popularity of traditional medicine and natural drugs， especially in Southeast Asia， the quality of traditional Chinese medicines （TCMs） has attracted the attention of regulators. China’s major TCM export destinations， such as South Korea， Japan， and Europe， have formulated strict maximum residue limits （MRLs） of pesticides in TCMs. Therefore， a sensitive and high-throughput method for the simultaneous determination of 101 pesticide residues in *Platycodonis radix* and extracts of *Angelica sinensis* was established， involving gel permeation chromatography （GPC） coupled with gas chromatography-ion trap mass spectrometry （GC-ITMS）. In this method， the samples were first ground into fine powder and extracted twice with 20 mL acetonitrile in an ultrasonic cleaner for 30 min. After centrifugation for 10 min at 6000 r/min， the supernatants were combined and dried at 40 ℃ using a rotary vacuum evaporator. The residue was re-dissolved in 2 mL ethyl acetate-cyclohexane （1∶1， v/v） and purified by gel permeation chromatography using a 40 cm×20 mm column. The eluent collecting time was optimized as 17-30 min to ensure both the recovery of target compounds and the removal of interferences such as pigments and lipids from the target compounds. The eluent was then dried and re-dissolved with 1 mL toluene for analysis. The 101 pesticide residues were separated using the DB-5MS capillary column and analyzed by ion trap mass spectrometry. The pretreatment conditions and ion trap mass spectrometry parameters were optimized to effectively reduce the interference of complex TCM matrices and greatly improve the quantitative accuracy of the analysis and recovery of the target pesticides. Three spiked levels of 101 pesticides were tested. The average recovery range was 58.3%-108.9% and the relative standard deviations （RSDs） at the three spiked levels （*n*=10） ranged from 0.4% to 16.5%. The limits of detection （LODs， *S/N*=3） of the 101 pesticide compounds ranged from 0.2 to 40.0 μg/kg， while the limits of quantification （LOQs， *S/N*=10） ranged from 0.6 to 120.0 μg/kg， which met the maximum residue limits of China’s main TCM export countries and organizations. This rapid analysis method was easy to operate and high throughput， with strong sensitivity and good repeatability. The employment of gel permeation chromatography overcame the drawback of inadequate cleanup of the solid phase extraction column during TCM analysis. The application of ion trap technology further eliminated the interference of matrix impurities and increased the accuracy of the quantitative and qualitative analyses. This method fills the knowledge gap in multiple pesticide residue determination in TCMs using gas chromatography-ion trap mass spectrometry and is a useful and beneficial alternative to current analytical methods of TCMs.

中药材是我国传统医学中的重要组成部分，也是中华民族传统文化的瑰宝，随着世界天然药物的研发热潮和中药在此次新冠疫情中的广泛应用，东南亚及欧美市场对中药的关注日益增强。目前，常用中药材多采用农业种植的方式获得，为防治作物病除害，不可避免在中药材的生产过程中使用农药，因此容易产生农药残留问题^[1]^。《中国药典》对农残检测方法的多次更新和修订正是为了不断增强中药材中农药残留的监管水平，促进中药材的质量提升和保障消费者的使用安全。2020年版《中国药典》一部规定了人参、甘草、西洋参等常用中药材中有机氯类农药残留检测方法及限量标准，其中五氯硝基苯（PCNB）、六氯苯、氯丹（顺式氯丹、反式氯丹和氧化氯丹之和）、六六六（BHC）不得超过0.1 mg/kg，七氯（七氯、环氧七氯之和）不得超过0.05 mg/kg，滴滴涕（DDT）不得超过1 mg/kg^[2]^；四部“2341农药残留量测定法”中给出了农药残留的检测标准和检出限，“0212药材和饮片检定通则”中规定了33种禁用农药，并且要求该类农药在中药材及饮片（植物类）中不得检出^[3]^。2017版《日本药局方》中规定了六六六和滴滴涕在20种药材中农药残留限量为0.2 mg/kg；《美国药典》规定了76种农药残留限量，《欧洲药典》在农药残留限量上与美国药典保持一致^[4]^；而韩国在第10版《韩国药典》（KP 10）中规定了生药及其萃取物中农药残留的限量，要求所有标准范围内的生药及其萃取物都需满足农药残留的限量要求，其中六六六限量为0.2 mg/kg，滴滴涕限量为0.1 mg/kg，狄氏剂、异狄氏剂、艾氏剂限量均为0.01 mg/kg^[5]^。

目前，中药材中农药残留检测多关注六六六、滴滴涕以及五氯硝基苯等有机氯农药和对硫磷、甲基对硫磷、甲胺磷、久效磷等有机磷农药^[6]^，崔丽丽等^[7]^利用QuEChERS-气相色谱-质谱联用法对人参中的六六六、滴滴涕和六氯苯等有机氯农药进行了检测，发现人参样品中*α*-BHC、*δ*-BHC、六氯苯和*p*，*p*'-滴滴伊均有检出，分别为0.011、0.014、0.007和0.018 mg/kg，张雪辉等^[8]^对23种中药材中六六六、滴滴涕和五氯硝基苯残留量进行了研究，结果发现大部分样品都有不同程度的检出，但农药残留量多在中国药典的限量范围之内。王芳焕等^[9]^用QuEChERS-气相色谱-串联质谱法对枸杞中20种农药残留进行了分析，在选取的20个样品中氯氟氰菊酯、哒螨灵、苯醚甲环唑和氯氰菊酯的检出率分别为30%、25%、35%、30%，其中氯氟氰菊酯的检测值最高为1.759 mg/kg，吕盼等^[10]^以气相色谱-串联质谱法检测了陈皮原料药材中179种农药，在选取的11批次样品中检出毒死蜱、戊唑醇、炔螨特、三氯杀螨醇、丙溴磷等农药，甚至个别样品检出的戊唑醇残留高达47.025 mg/kg。从检测方法上看，文献中对中药中农残的检测多采用色谱法并配以不同检测器^[11⇓-13]^，这些选择性检测器虽然灵敏度较高，但不能提供农药的结构信息，定性能力较弱，而采用色谱-质谱联用技术可以同时对各种类型的农药进行定性定量分析，以提高分析的准确性，常用的有气/液相色谱-质谱联用法和气/液相色谱-三重四极杆质谱法等^[14⇓⇓⇓⇓⇓⇓⇓-22]^， 2020版《中国药典》（四部）^[3]^“2341农药残留量测定法”中也新增了“第四法 农药多残留测定法（质谱法）”，提供了QuEChERS结合气相色谱-串联质谱法对中药中88种农药残留定量检测的方法，其净化过程采用分散固相萃取剂（无水硫酸镁900 mg、*N*-丙基乙二胺（PSA）300 mg、十八烷基硅烷键合硅胶300 mg、硅胶300 mg、石墨化炭黑90 mg）净化样品提取液。鲜见使用离子阱质谱法的相关报道。因此，本文建立了一种利用气相色谱-离子阱质谱技术检测中药材中101种常见农药残留的检测方法，在采用凝胶渗透色谱（GPC）净化的条件下，通过优化离子阱质谱参数，达到进一步排除复杂基质干扰、提高目标化合物信号的效果，其对复杂中药基质的净化效果和净化容量优于QuEChERS法，同时可采用凝胶渗透色谱设备实现高通量自动化前处理，为中药材的农药残留检测提供有益的方法补充。

## 1 实验部分

### 1.1 仪器、试剂与材料

气相色谱-离子阱质谱联用仪（AS 3000， TraceGC Ultra， PolarisQ MS，美国Thermo公司）；凝胶渗透色谱仪（美国J2 Scientific公司）； KQ-250型超声波清洗器（江苏昆山超声仪器有限公司）； EYELA-1000旋转蒸发仪（日本东京理化器械株式会社）； DSY-II自动快速浓缩仪（北京金科精华苑科技研究所）；丙酮、甲苯、乙酸乙酯、环己烷、二氯甲烷均为色谱级（美国J. T. Baker公司）； 101种农药标准品（德国Dr. Ehrenstorfer公司）； FW400A型高速万能粉碎机（北京科伟永兴仪器有限公司）。

标准溶液的配制：将各标准品分别以甲苯配制成1000 mg/L的标准储备液。根据气相色谱保留时间将101种农药分为两组（见表1），并分别按灵敏度高低用甲苯将单标储备液配制成两组混合标准储备液，实验时用甲苯将上述混合标准储备液稀释，配制成系列混合标准工作溶液。上述标准储备液置于-20 ℃冰箱避光保存。

**表1:** 101种农药残留的保留时间、质谱参数和在混合标准储备液中的浓度

Pesticide	Retention time/min	Precursor ion（*m/z*）	Product ion（*m/z*）	Excitation voltage/V	*C*/ （mg/L）
Group Ⅰ					
Methamidophos	5.79	141	95，126	1.0	4
Mevinphos	7.03	127	109	1.0	2
Methacrifos	8.16	208	180	1.2	1
Tecnazene	9.66	215	179	1.2	2
Phorate	11.64	231	203	1.0	2
Hexachlorobenzene	11.91	284	249	2.0	1
*β*-Hexachlorocyolohexane（*β*-BHC）	13.26	181	145，146	1.3	1
Terbufos	13.62	231	175，203	1.0	2
Fonofos	13.75	137	109	1.0	2
Diazinon	14.03	179	137，164	1.5	1
Chlorothalonil	14.06	266	168，170，231	2.0	4
*δ*-Hexachlorocyolohexane（*δ*-BHC）	14.60	219	181，183	1.3	1
Dichlofenthion	16.17	279	223，251	1.0	2
Parathion-methyl	16.87	263	246	1.0	4
Heptachlor	17.17	272	235，237	1.0	4
Bis（2，3，3，3-tetrachloropropyl）ether（S421）	17.90	132	117	1.0	5
Fenitrothion	18.50	277	260	1.0	2
Malathion	19.33	173	127	1.0	4
Chlorpyrifos	19.55	314	258，286	1.2	1
Triadimefon	21.43	208	180，181	0.8	2
Trichloronat	20.60	297	269	1.0	2
Isodrin	21.27	191	178	1.0	1
Isofenphos-methyl	21.61	199	121，167	1.0	1
Allethrin	22.91	123	81	1.0	4
Quinalphos	23.06	156	129	1.0	1
Procymidone	23.17	283	255	1.0	10
Paclobutrazol	24.56	236	125	0.7	4
*α*-Endosulfan	24.65	195	159，160	1.5	5
Isoprothiolane	26.58	204	118	0.5	2
Dieldrin	26.70	263	193，228	2.0	2
Carboxin	27.43	235	143，218	1.0	2
1-（2-Chlorophenyl）-1-（4-chlorophenyl）-2，2-dichloroethane（*o*，*p*'-DDD）	27.22	235	165，199	1.0	1
Kresoxim-methyl	28.11	206	116，132	1.2	3
Chlorfenapyr	28.47	247	227	2.0	3
*β*-Endosulfan	29.23	195	159，160	1.5	5
1，1-Dichlor-2，2-bis（4-chlor-phenyl）ethane（*p*，*p*'-DDD）	30.16	235	165，199	1.0	1
Ethion	30.56	231	175，203	1.2	2
Edifenphos	32.42	310	186，201	1.0	2
Resmethrin	35.55	171	128，143	1.0	3
Ethyl-*p*-nitrophenyl phenylphosphonothioate（EPN）	37.33	169	141	1.0	2
Dicofol	38.04	139	111	1.2	3
Tebufenpyrad	39.19	333	276	1.0	4
Phosalone	40.14	182	111，138	1.0	2
*λ*-Cyhalothrin	41.68	181	152	1.7	1
Permethrin	42.79	183	168	1.2	2
Cyfluthrin	43.67	206	150，151	2.0	4
*β*-Cypermethrin	44.05	181	152	1.5	4
Fenvalerate	44.82	225	119，147	1.0	3
*τ*-Fluvalinate	45.02	250	200	1.3	3
Difenoconazole	45.39	323	265	1.0	3
Deltamethrin	45.85	181	152	2.0	4
Pesticide	Retention time/min	Precursor ion（*m/z*）	Product ion（*m/z*）	Excitation voltage/V	*C*/ （mg/L）
Group Ⅱ					
Dichlorvos	5.86	185	93	1.0	4
Acephate	7.41	136	94	1.0	10
Omethoate	9.62	156	110，141	1.0	10
Ethoprophos	10.43	158	114，130	1.0	3
*α*-Hexachlorocyolohexane（*α*-BHC）	11.78	181	145，146	1.0	1
Dimethoate	12.38	125	79	1.0	4
*γ*-Hexachlorocyolohexane（*γ*-BHC）	12.93	181	145，146	1.0	2
Quintozene	13.00	237	141，143	1.6	2
Pyrimethanil	14.14	198	183	1.7	3
Disulfoton	14.49	245	217，189	1.0	5
Tefluthrin	14.90	177	127	1.2	1
Iprobenfos	15.37	204	171	1.0	3
Acetochlor	16.47	223	132，146	1.0	4
Chlorpyrifos-methyl	16.47	286	208，271	1.2	4
Alachlor	16.98	188	160	1.0	3
Fenchlorphos	17.52	285	270	1.2	2
Pirimiphos-methyl	19.58	290	151，233，262	1.0	3
Fenthion	19.89	278	135，245	1.2	1
Parathion-ethyl	20.13	291	142，263	1.0	4
Isocarbofos	20.43	136	108	0.8	3
Bromophos-methyl	20.92	331	316	1.0	1
Pirimiphos-ethyl	21.41	333	180	1.0	2
Isofenphos	22.73	213	185	1.0	2
Ethychlozate	22.78	165	110，138	1.0	3
Phenthoate	23.10	274	121，246	1.0	2
Methidathion	23.95	145	85	0.8	2
2，2，*o*，*p*'-Tetrachlorovinylidenebisbenzene（*o*，*p*'-DDE）	24.25	246	176，211	2.0	1
Ditalimfos	25.28	243	130，148	1.5	2
Profenofos	26.66	339	269，297，311	1.0	4
1，1-Dichloro-2，2-bis（4-chlorophenyl）ethene（*p*，*p*'-DDE）	26.80	246	176	2.0	1
Myclobutanil	27.38	179	125	1.0	2
Flusilazole	27.59	233	165	1.4	3
Endrin	29.23	263	193，228	2.0	5
2，4'-Dichlorodiphenyltrichloroethane（*o*，*p*'-DDT）	30.17	235	165，199，200	1.0	2
Triazophos	31.81	257	162	1.0	3
1，1'-（2，2，2-Trichloroethyliden）bis-（4-chlorobenzol）（*p*，*p*'-DDT）	33.14	235	165，199，200	1.0	2
Tebuconazole	34.35	250	125，163	1.2	3
Diflufenican	35.36	266	216，238	2.0	3
Fenpropathrin	38.83	181	152	2.0	5
Bifenthrin	39.19	181	165，166	0.9	2
Leptophos	40.11	377	269，362	1.0	2
Azinphos-methyl	40.20	132	77，104	0.8	2
Acrinathrin	42.18	181	152	2.0	2
Coumaphos	42.82	362	226，306，334	1.0	2
Cypermethrin	43.92	181	152	1.8	3
Flucythrinate	44.01	225	181	1.0	5
Esfenvalerate	44.83	225	119，147	1.0	3
Flumioxazin	44.92	354	312，326	1.0	2
Indoxacarb	45.67	264	232	0.8	3
Tralomethrin	45.83	181	152	2.0	4

柴胡、桔梗（北京同仁堂中药进出口有限公司）经粉碎机粉碎成60~200目粉末，低温、干燥保存。当归提取物（河南中冠健业生物科技有限公司）。

### 1.2 实验条件

#### 1.2.1 色谱条件

DB-5MS毛细管色谱柱（30 m×0.25 mm×0.25 μm）；载气：高纯氦气（纯度≥99.999%）；流速：1 mL/min；进样量：1 μL；进样方式：不分流进样；进样口温度：250 ℃；柱起始温度40 ℃，保持1 min，以30 ℃/min升到160 ℃，以2 ℃/min升到230 ℃，以20 ℃/min升到300 ℃，保持5 min。

#### 1.2.2 质谱条件

电离方式：电子轰击源（EI）；电离能量：70 eV，离子源温度250 ℃，传输线温度：280 ℃，最大激发能量（maximum excitation energy， *q*）： 0.3。采用二级离子监测模式：每种化合物选择一个母离子，然后选择1~3个子离子，每种化合物的母离子、子离子、扫描时间、碰撞能量等见表1。

### 1.3 提取与净化

称取5.0 g粉碎好的试样，加入20 mL乙腈超声30 min， 6000 r/min离心5 min后转移提取液到鸡心瓶中，试样残渣用20 mL乙腈按上述步骤再提取一次，合并两次提取液并在40 ℃以下旋转蒸发浓缩至近干，用2 mL乙酸乙酯-环己烷（1∶1， v/v）洗涤旋转蒸发瓶，经0.45 μm滤膜过滤转移至GPC进样瓶。

GPC条件为：柱长40 cm，内径20 mm，填料粒度200~400目，流动相乙酸乙酯-环己烷（1∶1， v/v），进样量2 mL，流速4 mL/min，收集时间17~30 min。收集后，在40 ℃以下浓缩至近干。用1 mL甲苯定容，待检测。

## 2 结果与讨论

### 2.1 色谱条件的选择

为提高检测灵敏度，让化合物尽量实现最大分离，尝试了不同的程序升温条件。本实验测定的101种农药的出峰温度主要集中在160~230 ℃，比较了该温度范围内升温速率为5 ℃/min（见图1a）和2 ℃/min（见图1b）时化合物的分离效果，可以看出后者能实现更好的分离效果以减少化合物的共流出对质谱检测灵敏度的负面影响，因此采用160~230 ℃间升温速率为2 ℃/min作为程序升温的中段条件。

**图1:**
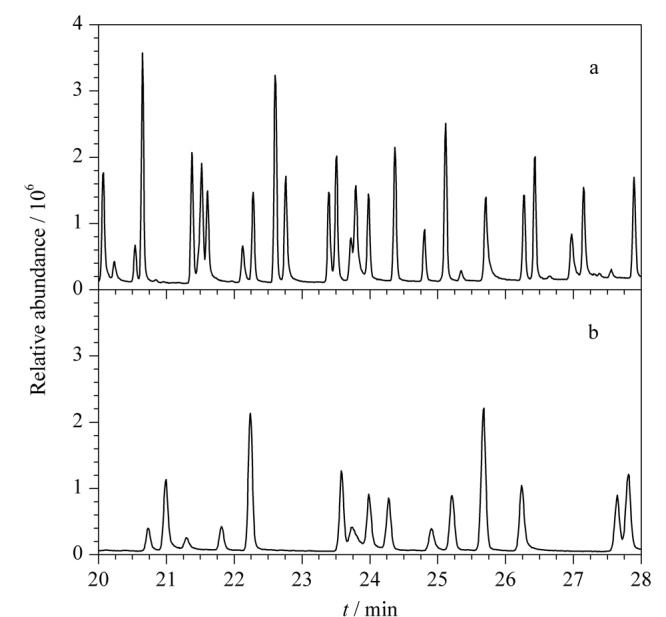
升温速率为（a）5 ℃/min和（b）2 ℃/min时色谱分离效果对比

离子阱质谱是时间串联型质谱，离子被抓取和裂解时都在同一个阱中完成，实验发现当同一保留时间有多个化合物时仪器检测灵敏度会略低，因此，根据101种农药的出峰时间和分离度，将其分为Ⅰ组和Ⅱ组进行分析，能获得更好的灵敏度，空白桔梗基质添加混合标准溶液的二级总离子流图见图2。

**图2:**
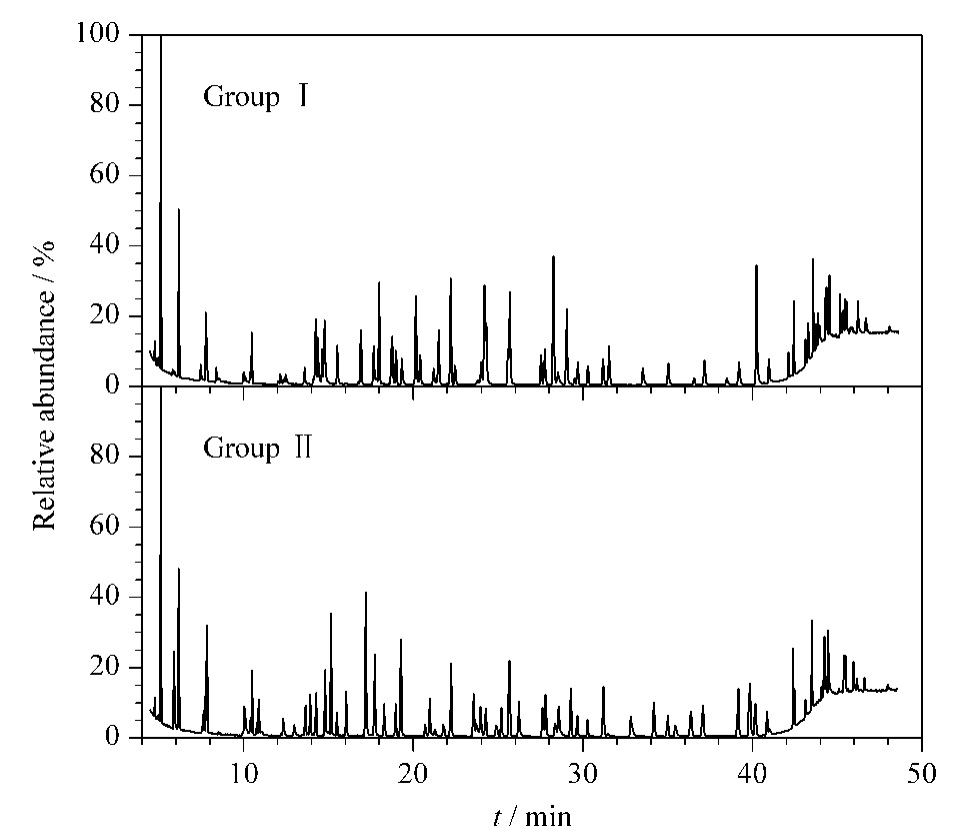
空白加标桔梗溶液的总离子流图

### 2.2 质谱参数的优化

二级质谱的定性定量通常是每种化合物选择一个母离子，采用合适的碰撞电压对其进行二级电离，然后在碎片离子中选择1~3个碎片离子对目标化合物定性定量，这符合欧盟指令2002/657/EC规定的在应用质谱仪对目标化合物进行确证时的评价准则。在本方法中，中药样品基质非常复杂，具有很强的基质干扰，因此选择满足条件且基质干扰较小的母离子、选择合适的碰撞电压及最大激发能量就成为优化质谱参数的关键问题。

首先，选择合适的母离子和子离子是保证目标化合物获得最佳灵敏度的重要前提。母离子应优先选择*m/z*较大且相对丰度较大的碎片离子，但由于某些*m/z*或丰度较大的离子有基质干扰，因此需要选择其他更合适的离子作为母离子以避免干扰，例如，乙拌磷的初级碎裂质谱图中（见图3a）， *m/z* 186的碎片离子丰度明显高于*m/z* 245，本应选择*m/z*为186的碎片离子作为母离子，但因为桔梗样品中也产生*m/z* 186碎片离子，采用此离子作为母离子时样品基质产生的背景干扰（见图3b）比*m/z*为245的碎片离子作为母离子时（见图3c）高10倍，所以为降低化合物电离时的基质效应，最终选择*m/z*为245的碎片离子作为母离子。

**图3:**
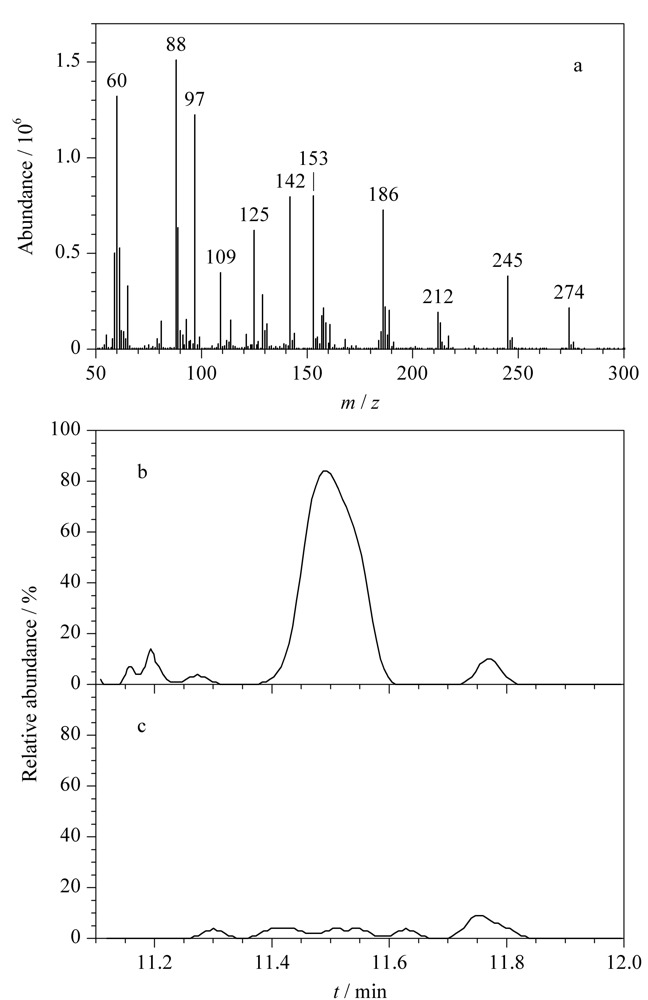
（a）乙拌磷的全扫质谱图及选用（b）*m/z* 186和（c）*m/z*245为母离子时空白桔梗基质的总离子流图

其次，一般来说提高激发电压可使离子阱中的母离子加速与惰性气体分子发生剧烈碰撞，促进母离子的裂解，即碰撞电压的大小能影响母离子的裂解程度。通常选择能使母离子充分裂解的电压以确保化合物二级质谱的响应达到最大，但不同化合物裂解情况不一，如降低杀扑磷激发电压反而可以增加子离子的丰度，当激发电压由1 V降低至0.8 V时子离子丰度增加了43.4%（见图4a）。又如溴苯磷的激发电压由1 V增加到1.5 V时，虽然可以使其母离子*m/z* 377碎裂更完全，可子离子*m/z* 362的丰度也随之降低36.3%，因此仍然采用1 V作为激发电压（见图4b）。

**图4:**
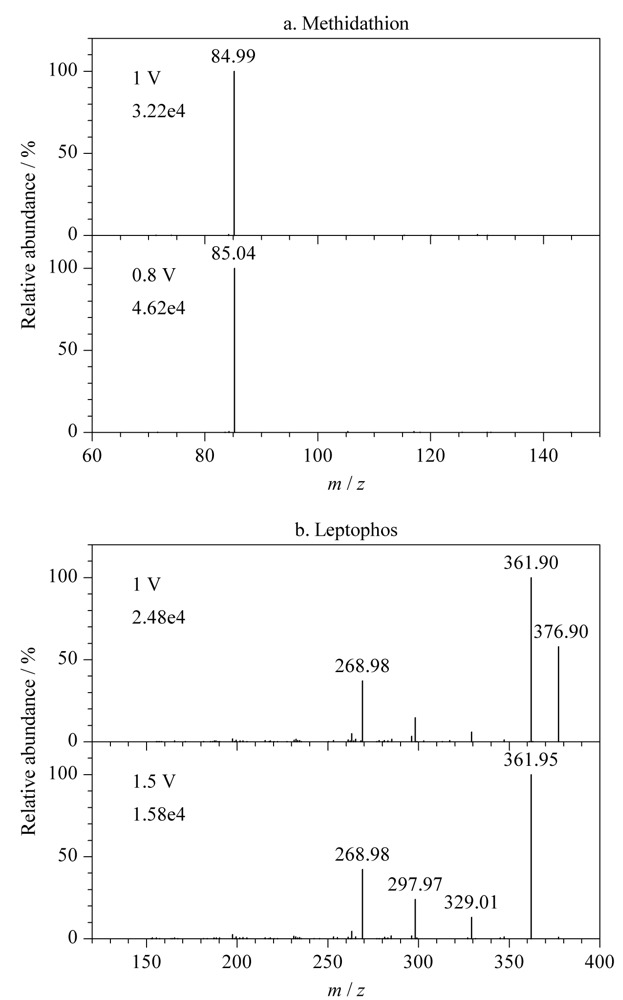
不同激发电压下（a）杀扑磷和（b）溴苯磷的母离子碎裂质谱图

最后，离子阱的特征是选择和储存离子，最大激发能量（*q*值）是表征离子在阱中稳定性的无量纲参数。*q*值大，离子在阱中的振荡能量高，离子易裂解，但同时也会造成碎片离子易从阱中抛出。相反，*q*值小，离子在阱中相对稳定，但母离子不易裂解，相应的子离子产率比较低。本实验发现调节*q*值为0.225、0.3、0.45时对多数母离子碎裂的影响不大，因此全部采用0.3，这也与Derouiche等^[23]^的研究结果一致。

### 2.3 前处理条件的选择

考虑到对多种农药残留的整体提取效率，重点考察了乙腈、丙酮和乙酸乙酯等3种常用于多残留检测的提取溶剂，经实验对比，3种溶剂虽然在个别农药上的提取效率各有高低、但综合来看乙腈对多种农药成分的整体提取效率略好于另外两种，目标检测物的响应值较高，同时提取的色素和油脂等杂质也相对较少，这有利于降低背景干扰，提高检测灵敏度，因此选定乙腈作为提取溶剂。

净化方式对去除杂质、降低背景干扰、提高方法检出限具有重要意义，实验重点考察了各种常用SPE固相萃取小柱和GPC的净化效果。由于中药材基质复杂、脂类杂质较多，经过对比碳十八固相萃取柱（C_18_）、弗罗里硅土固相萃取柱（Florisil）、中性氧化铝固相萃取柱（N-Al_2_O_3_）以及GPC净化后的效果（见图5），净化效果从总离子流图上判断依次为GPC>Florisil≈N-Al_2_O_3_>C_18_。因此，决定采用GPC净化。

**图5:**
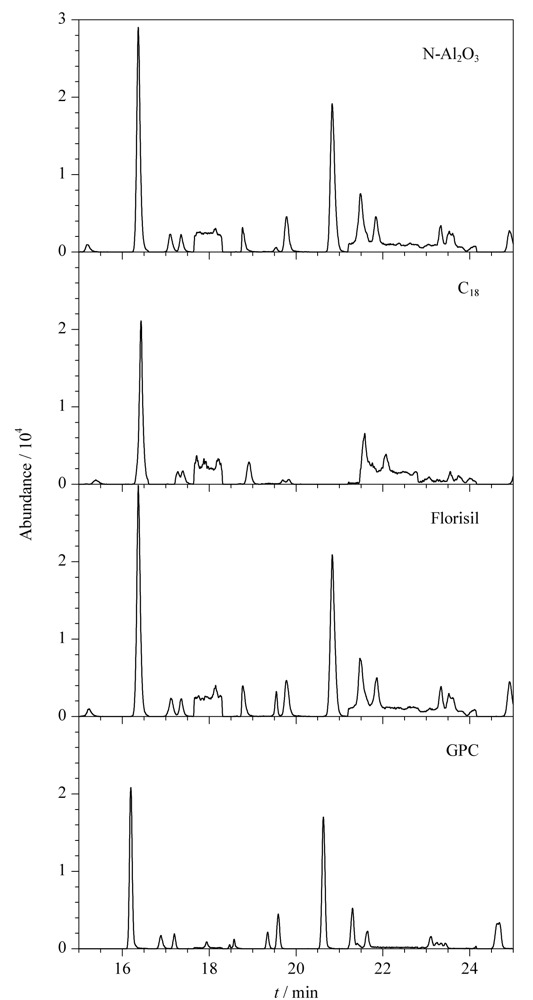
不同净化方法下空白加标桔梗基质的总离子流图

### 2.4 线性范围、定量限和回收率

本实验采用空白样品提取液配制基质匹配标准曲线，以各农药的质量浓度（*X*， μg/L）及该化合物峰面积（*Y*）绘制标准曲线，得到线性方程和相关系数（*r*^2^），见附表1和附表2（详见https：//www.chrom-China.com）。结果表明，101种农药在一定线性范围内呈良好的线性关系，相关系数>0.995，满足分析要求。以基质空白标准溶液*S/N*≥3和10分别计算得到方法的检出限（LOD）和定量限（LOQ）， 101种农药的检出限和定量限范围分别为0.2~40.0 μg/kg和0.6~120.0 μg/kg，具体数值见附表1和附表2。

分别对桔梗原药和当归提取物进行了高、中、低3水平添加试验（*n*=10），计算回收率和精密度。测定结果表明，101种农药在这两种基质中的平均回收率为58.3%~108.9%， 10次平行测定的相对标准偏差为0.4%~16.5%，结果均满足定量分析要求，每种基质中各农药的添加水平和回收率等测定结果详见附表1和附表2。

### 2.5 实际样品检测

应用本方法检测了本市药店购买的3批桔梗和3批当归原药，未检测到样品中含有本方法相关农药，随样品进行基质添加，其回收率满足方法要求。通过对当归原药基质添加的方法，采用本方法和《桑枝、金银花、枸杞子和荷叶中488种农药及相关化学品残留量的测定 气相色谱-质谱法》（GB 23200.10-2016）同时检测，相同农药定量结果的精密度小于15%，说明本方法可用于相关农药在中药材中的定量检测。

## 3 结论

本研究通过使用气相色谱-离子阱质谱技术建立了一种可操作性强、高效且可同时检测桔梗原药及当归提取物中101种农药残留的方法。本方法采用GPC为净化手段，有效去除了样品中的油脂、色素等干扰仪器检测的杂质，采用气相色谱-离子阱质谱二级离子模式检测，通过对色谱条件、质谱扫描参数的优化，进一步排除了基质干扰。通过对比桔梗原药及当归提取物的分析结果发现，相对于杂质含量较少的提取物，原药中绝大部分农药的回收率和灵敏度没有明显差异，说明本方法的净化手段和检测技术能有效排除复杂中药基质中杂质的影响，实现了同时对101种农药的高效、高灵敏度定性和定量分析，方法可满足当前韩国、日本、欧盟规定的相关农药最大残留限量要求，是对现有中药材中多农残检测方法的有益补充。
